# Bevacizumab suppressed degenerative changes in articular cartilage explants from patients with osteoarthritis of the knee

**DOI:** 10.1186/s13018-023-03512-2

**Published:** 2023-01-10

**Authors:** Masaichi Sotozawa, Ken Kumagai, Kimi Ishikawa, Shunsuke Yamada, Yusuke Inoue, Yutaka Inaba

**Affiliations:** Department of Orthopaedic Surgery, Graduate School of Medicine, Yokohama City University, 3-9 Fukuura, Kanazawa-ku, Yokohama, 236-0004 Japan

**Keywords:** Articular cartilage, Bevacizumab, Osteoarthritis, Protection, Vascular endothelial growth factor

## Abstract

**Background:**

This study was designed to test the hypothesis that blockade of vascular endothelial growth factor (VEGF) suppresses degenerative changes in articular cartilage from patients with osteoarthritis (OA).

**Methods:**

Articular cartilage from eight OA patients was subjected to explant culture for 2 days in the presence or absence of 10 ng/ml recombinant interleukin (IL)-1β. The blocking effect of VEGF was examined by the addition of 10 or 100 ng/ml of bevacizumab. The culture media were harvested, and markers for cartilage degradation were measured by sandwich enzyme-linked immunoassay. Total RNA was isolated from cartilage tissues, and gene expressions associated with the anabolic response were examined by the quantitative real-time polymerase chain reaction.

**Results:**

Bevacizumab significantly reduced concentrations of matrix metalloproteinase (MMP)-2, MMP-3, and cartilage oligomeric matrix protein in the culture media with and without IL-1β. Significant suppressive effects of bevacizumab on MMP-9 and MMP-13 were shown only in the presence of IL-1β. Gene expression of Col2a1 was significantly increased by the addition of bevacizumab in the absence of IL-1β.

**Conclusion:**

Bevacizumab inhibits catabolic reactions and stimulates anabolic function in articular cartilage derived from OA patients directly, suggesting a protective effect on articular cartilage from OA progression.

## Introduction

Osteoarthritis (OA) is a common degenerative joint disease characterized by progressive loss of articular cartilage. Despite numerous research efforts to elucidate mechanisms of OA pathophysiology, there are still no established, clinically used, disease-modifying OA drugs (DMOADs). As one of the pathological mechanisms, angiogenesis is known to play an important role in the initiation and progression of OA, and it may be a potential therapeutic target for OA [1].

Vascular endothelial growth factor (VEGF) is a potent angiogenic factor and a critical mediator of endochondral ossification, which is involved in an essential process of skeletal development and growth [2]. However, it has a distinct role in postnatal homeostasis of articular cartilage, and VEGF is undetectable in healthy articular cartilage [3]. In contrast, expressions of VEGF and its receptors are found in the articular cartilage of OA patients [4]. Therefore, aberrant expression of VEGF in the articular cartilage may contribute to the pathogenesis of OA [5].

Anti-VEGF therapy is an attractive option as a potential OA treatment. Bevacizumab is a commercially available anti-VEGF monoclonal antibody currently used for the treatment of solid cancers, age-related macular degeneration, and diabetic retinopathy. Intra-articular injection of bevacizumab reduced articular cartilage degeneration, with less osteophyte formation and synovitis in a rabbit OA model [6]. Intra-articular injection of bevacizumab prevented joint inflammation with reduced angiogenesis, inhibited synovial proliferation, and reduced VEGF and MMP-1 expressions in a rabbit OA model [7]. However, the effect of anti-VEGF therapy on human cartilage degeneration was not directly investigated, and it needs to be elucidated. Although it seems difficult to conduct a clinical evaluation, assessment of the direct effect of anti-VEGF drugs on human articular cartilage is possible using articular cartilage samples derived from primary OA patients who underwent knee arthroplasty.

It was hypothesized that VEGF blockade suppresses degenerative changes in human articular cartilage. In this study, the effects of anti-VEGF agents on human articular cartilage explants from OA patients were examined.

## Materials and methods

### *Ex vivo* cartilage explant culture

Cartilage tissues of the lateral femoral condyle were obtained from eight patients (age 70.1 ± 8.2 years) with OA (Kellgren–Lawrence grade 4) of the medial compartment who underwent total knee arthroplasty, with informed consent and approval of the institutional review board at Yokohama City University (#B191100008). After subchondral bone was trimmed out, cylindrical, full-depth, articular cartilage explants were harvested using an osteochondral harvester with an internal diameter of 6.5 mm (Smith & Nephew Inc., Andover, MA, USA), and they were cultured in serum-free media for 24 h. Cartilage explants were then cultured in Dulbecco’s modified Eagle’s medium with high glucose supplemented with 10% fetal bovine serum and 1% penicillin/streptomycin for 2 days. All cultures were performed at 37 °C in a humidified atmosphere containing 5% CO_2_. Cartilage explants were also cultured with the addition of 10 ng/ml recombinant interleukin (IL)-1β (PeproTech, Rocky Hill, NJ, USA), as previously described [8–10]. The blocking effect of VEGF was examined by the addition of 10 or 100 ng/ml of bevacizumab (Avastin, Chugai Pharmaceutical Co., Ltd, Tokyo, Japan).

### Enzyme-linked immunosorbent assay (ELISA)

The supernatants of cartilage explants were analyzed with ELISA using commercially available test kits (Quantikine, R&D Systems, Minneapolis, MN, USA) according to the manufacturer’s instructions. The concentrations of the following cartilage degradation markers were measured: matrix metalloproteinase (MMP)-2, MMP-3, MMP-9, MMP-13, tissue inhibitor of metalloproteinase (TIMP)-1, TIMP-2, and cartilage oligomeric matrix protein (COMP). Sample dilution and validation of assays are summarized in Table 1.

**Table 1:** Sample dilution and validation of immunosorbent assay

Biomarkers	Manufacture/catalog#	Dilution	Intra-assay CV (%)	Inter-assay CV (%)
MMP-2	R&D/MMP200	1:10	5.7	8.4
MMP-3	R&D/DMP300	1:1000	6.3	8.5
MMP-9	R&D/DMP900	1:50	4.4	10.1
MMP-13	R&D/DY511	1:10	3.5	7.3
TIMP-1	R&D/DTM100	1:100	3.7	6.8
TIMP-2	R&D/DTM200	1:100	6.4	11.2
COMP	R&D/DCMP0	1:500	2.8	5.8

*CV* Coefficient of variation; *MMP* Matrix metalloproteinase; *TIMP* Tissue inhibitor of metalloproteinase; *COMP* Cartilage oligomeric matrix protein

### Total RNA isolation and real-time polymerase chain reaction (PCR)

As described in detail previously [11], mRNA expression levels were analyzed using the following procedures. Total RNA was isolated from a homogenized cartilage fragment using TRIzol reagent (Invitrogen, Carlsbad, CA, USA). RNA was quantified by measuring absorbance at 260 nm, and the quality was assessed by determining the 260/280 nm absorbance ratio. First-strand cDNA synthesis was performed with 0.5 μg or 1 μg of total RNA in a total volume of 20 μl using an iScript™ advanced cDNA synthesis kit (BIO-RAD, Richmond, CA, USA). Quantitative real-time PCR was carried out using TaqMan gene expression assays (Applied Biosystems, Foster City, CA, USA) on a CFX96TM real-time PCR detection system (BIO-RAD) in a 20-μl reaction volume. Expression of the gene of interest was normalized to GAPDH expression. TaqMan gene expression assays used in this study were as follows: *Col2a1* (Hs00264051_m1); *Col10a1* (Hs00166657_m1); *Sox9* (Hs1001343_g1); and *Runx2* (Hs01047973_m1).

### Statistical analysis

Statistical analysis was carried out using BellCurve for Excel version 4.02 (Social Survey Research Information, Tokyo, Japan). All data are presented as means and standard deviation. The nonparametric Kruskal–Wallis test was used to test for significant differences among the test groups. When a significant difference was detected, Steel’s post hoc test was performed to compare each of the treatments with a control. An adjusted *P* value < 0.05 was considered significant.

## Results

### Effect of bevacizumab on cartilage degradation

**Fig. 1:**
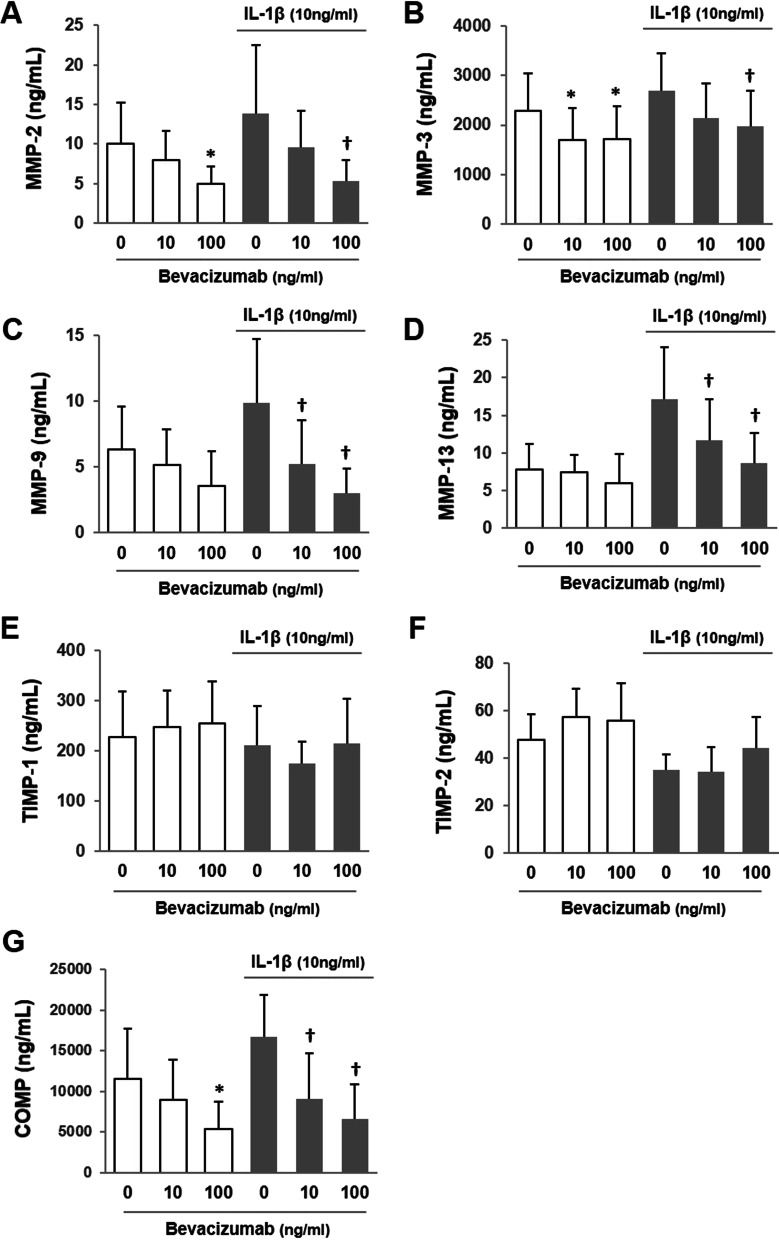
Effect of bevacizumab on cartilage degradation in the presence and absence of interleukin (IL)-1β. Concentrations of markers in culture media were measured by enzyme-linked immunosorbent assay. **A** Matrix metalloproteinase (MMP)-2, **B** MMP-3, **C** MMP-9, **D** MMP-13, **E** tissue inhibitor of metalloproteinase (TIMP)-1, **F** TIMP-2, and **G** cartilage oligomeric matrix protein (COMP). Values are given as means and standard deviation. *P < 0.05 versus IL-1β(-)/bevacizumab 0 ng/ml and †*P* < 0.05 versus IL-1β(+)/bevacizumab 0 ng/ml

To assess the effect of bevacizumab on cartilage degradation, concentrations of MMPs, TIMPs, and COMP were measured by ELISA. The concentration of MMP-2 was significantly reduced by the addition of 100 ng/ml bevacizumab (*P* < 0.05, vs. 0 ng/ml) both with and without IL-1β (Fig. 1A). The concentration of MMP-3 was significantly reduced by the addition of 10 or 100 ng/ml bevacizumab (*P* < 0.05, vs. 0 ng/ml) without IL-1β and 100 ng/ml bevacizumab (*P* < 0.05, vs. 0 ng/ml) with IL-1β (Fig. 1B). No significant changes in the concentrations of MMP-9 and MMP-13 under the culture condition without IL-1β were found with the addition of bevacizumab (Fig. 1C, D). In contrast, when the cartilage tissue was cultured with IL-1β, concentrations of MMP-9 and MMP-13 were significantly decreased by the addition of 10 or 100 ng/ml bevacizumab (Fig. 1C, D). No significant effect of bevacizumab was found on the concentrations of TIMP-1 and TIMP-2 under the culture conditions with and without IL-1β (Fig. 1E, F). The concentration of COMP was significantly reduced by the addition of 100 ng/ml bevacizumab (*P* < 0.05, vs. 0 ng/ml) without IL-1β and 10 or 100 ng/ml bevacizumab (*P* < 0.05, vs. 0 ng/ml) with IL-1β (Fig. 1G).

### Effect of bevacizumab on chondrogenesis-related markers

**Fig. 2:**
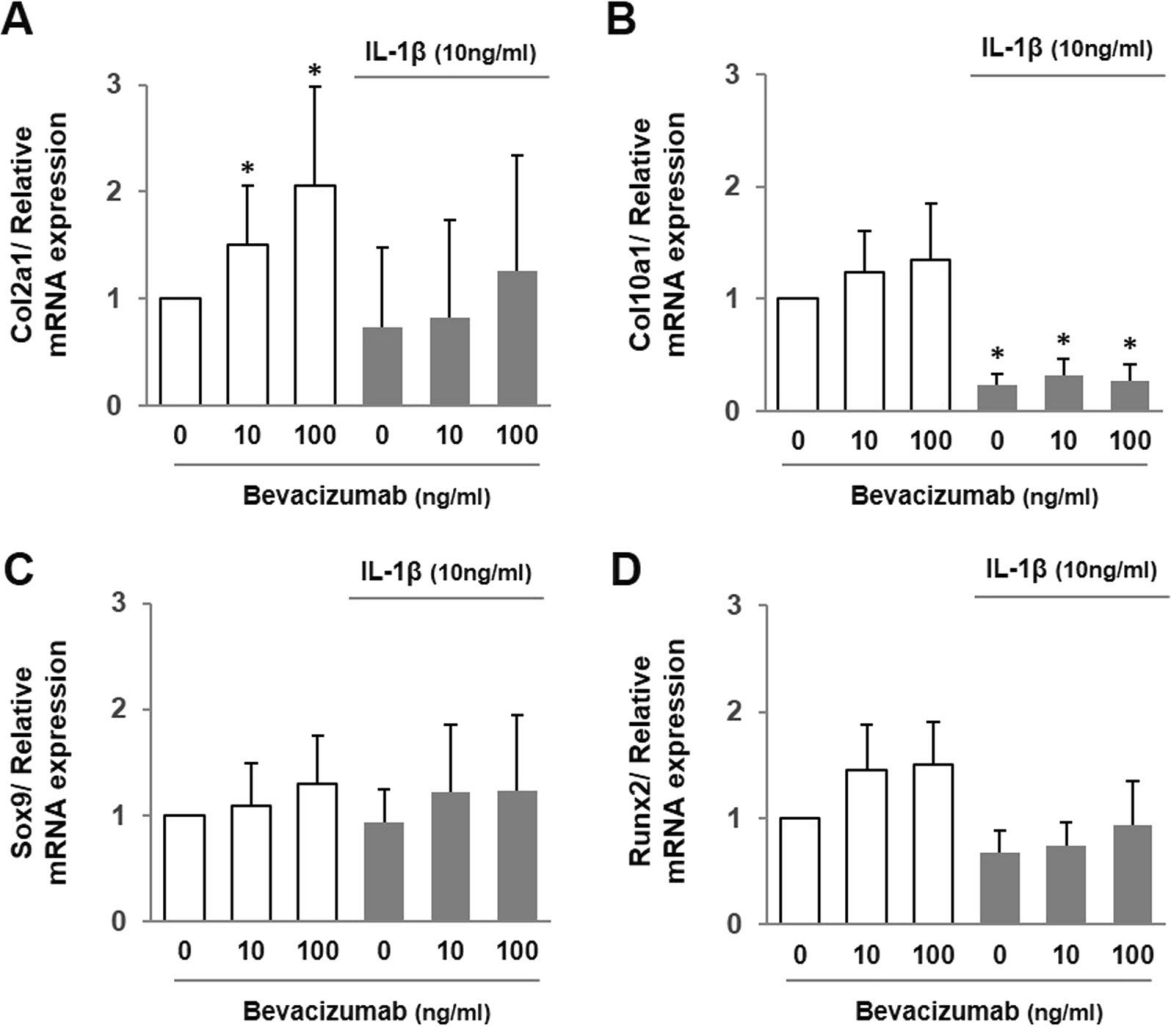
Effect of bevacizumab on cartilage metabolism in the presence and absence of interleukin (IL)-1β. Relative mRNA expressions were examined with quantitative real-time PCR. **A** Col2a1, **B** Col10a1, **C** Sox9, and **D** Runx2. Values are given as means and standard deviation. *P < 0.05 versus IL-1β(−)/bevacizumab 0 ng/ml and †*P* < 0.05 versus IL-1β(+)/bevacizumab 0 ng/ml

To assess the effect of bevacizumab on anabolic function in OA cartilage, gene expressions of Col2a1, Col10a1, Sox9, and Runx2 were analyzed. Relative mRNA expression of Col2a1 was significantly increased by the addition of 10 or 100 ng/ml of bevacizumab without IL-1β (*P* < 0.05, vs. 0 ng/ml), but no significant changes were found with IL-1β (Fig. 2A). Relative mRNA expression of Col10a1 was significantly reduced with IL-1β compared to the non-IL-1β control (*P* < 0.05), but no significant changes were found by the addition of bevacizumab (Fig. 2B). Relative mRNA expressions of Sox9 and Runx2 were slightly increased by the addition of bevacizumab, but these changes were not significant (Fig. 2C, D).

## Discussion

The most important findings of this study were that bevacizumab showed an inhibitory effect on catabolic reactions and a stimulatory effect on anabolic function in articular cartilage explants derived from OA patients. The results supported the initial hypothesis that VEGF blockade suppresses degenerative changes in human articular cartilage.

Various attempts have been made to develop DMORDs by using in vitro models, and preclinical evaluation has been carried out predominantly in animal models. Though OA is a chronic degenerative disease, and the majority of patients have no history of trauma, most experimental animal models are surgically induced, post-traumatic OA [12, 13]. Since there is a lack of detailed information about the effects of anti-VEGF therapy on the human *ex vivo* OA cartilage model, this study design was used.

VEGF is a well-studied angiogenic factor, and its involvement in OA pathogenesis has been widely investigated. Increased VEGF levels are associated with OA progression, and VEGF seems to play a key role in cartilage metabolism [1]. Several in vitro studies demonstrated the direct effect of VEGF signaling on the increase in pre-catabolic mediators in chondrocytes [4, 14]. Exogenous VEGF stimulated the increase in proinflammatory cytokines in chondrocytes [14]. The present study demonstrated that bevacizumab reduced the production of catabolic proteins in human OA cartilage explants. Furthermore, bevacizumab also showed an inhibitory effect on the catabolic response of cartilage under IL-1β exposure. Thus, bevacizumab therapy has a potential to protect against cartilage degradation in OA patients.

VEGF inhibits the anabolic function of articular chondrocytes. Rat articular chondrocytes cultured in a monolayer with VEGF showed reduced expressions of aggrecan and type II collagen [15]. The present study demonstrated the stimulatory effect of bevacizumab on the anabolic response in arthritic articular cartilage, suggesting recovery of anabolic function. A synergistic inhibitory effect on cartilage anabolism was found in combination with IL-1β [15]. The dual effects of VEGF and inflammatory cytokines on chondrocytes seem to be a part of mechanisms of cartilage degradation in osteoarthritis. In the present study, bevacizumab did not significantly affect anabolic responses in the cartilage explants under IL-1β exposure, suggesting reduced anabolic function caused by the inflammatory cytokine. In addition, expressions of Sox9 and Runx2 were not significantly changed by the addition of bevacizumab, although mean values were slightly increased. Physiological and pathological variability of human OA samples may have affected the relatively large standard deviation and the statistical results. However, the effect of bevacizumab on anabolic responses seems to be limited as compared to the effect on catabolic ones.

The process of endochondral ossification, including chondrocyte hypertrophy, production of proteinases, and chondrocyte apoptosis, is thought to play a key role in the initiation and progression of OA [16]. Modulation of the endochondral process in mature articular cartilage seems to be a potential target for OA treatment [11, 17]. Expression of VEGF and subsequent angiogenesis contribute to cartilage growth and endochondral ossification during growth plate development, but expression of VEGF is physiologically suppressed in adult cartilage, leaving mature cartilage avascular. Under pathological conditions such as inflammation or mechanical stress, VEGF is re-upregulated with the endochondral process in articular chondrocytes and involved in the initiation and progression of OA [5]. Thus, one of the hypothetical strategies for OA treatment is to inhibit pathological angiogenesis and endochondral process by anti-VEGF therapy.

Preclinical studies of anti-VEGF therapy for OA using animal models demonstrated the effects on various tissues including articular cartilage, subchondral bone, and synovial tissue [6, 7, 18, 19]. The present study investigated the direct response of the OA articular cartilage to an anti-VEGF agent without the influence of synovial tissue and confirmed the stimulation of anabolic function and suppression of catabolic reactions, suggesting protective effects on articular cartilage.

Although anti-VEGF therapy alone has potential efficacy for OA treatment, synergistic effects may be generated in combination with the other biologic therapies. For example, intra-articular injection of platelet-rich plasma (PRP) has been extensively used for OA treatment, and the combination with anti-VEGF is a potential treatment option. A recent study demonstrated that VEGF-attenuated PRP improves articular cartilage repair in a rat OA model [20]. Since PRP contains several growth factors including VEGF, blockade of VEGF function may augment the efficacy of PRP therapy.

## Conclusion

This study provides insights into the therapeutic potential of bevacizumab for OA. Bevacizumab inhibits catabolic reactions and stimulates anabolic function in articular cartilage derived from OA patients directly, suggesting that it has a protective effect on articular cartilage from progression of OA. Bevacizumab is a candidate DMOAD for the treatment of OA.

## Data Availability

The data and materials used and/or analyzed during the current study are not publicly available but available from the corresponding author upon reasonable request.
